# Divergent selection-induced obesity alters the composition and functional pathways of chicken gut microbiota

**DOI:** 10.1186/s12711-016-0270-5

**Published:** 2016-11-28

**Authors:** Jinmei Ding, Lele Zhao, Lifeng Wang, Wenjing Zhao, Zhengxiao Zhai, Li Leng, Yuxiang Wang, Chuan He, Yan Zhang, Heping Zhang, Hui Li, He Meng

**Affiliations:** 1 Department of Animal Science, School of Agriculture and Biology, Shanghai Jiao Tong University, Shanghai Key Laboratory of Veterinary Biotechnology, Shanghai, 200240 People’s Republic of China; 2 College of Animal Science and Technology, Northeast Agricultural University, Harbin, 150030 People’s Republic of China; 3 College of Food Science and Engineering, Inner Mongolia Agricultural University, Key Laboratory of Dairy Biotechnology and Engineering, Hohhot, 010018 People’s Republic of China; 4 Shanghai Personal Biotechnology Limited Company, Shanghai, 200231 People’s Republic of China; 5 Shanghai Animal Disease Control Center, Shanghai, 201103 People’s Republic of China

## Abstract

**Background:**

The gastrointestinal tract is populated by a complex and vast microbial network, with a composition that reflects the relationships of the symbiosis, co-metabolism, and co-evolution of these microorganisms with their host. The mechanism that underlies such interactions between the genetics of the host and gut microbiota remains elusive.

**Results:**

To understand how genetic variation of the host shapes the gut microbiota and interacts with it to affect the metabolic phenotype of the host, we compared the abundance of microbial taxa and their functional performance between two lines of chickens (fat and lean) that had undergone long-term divergent selection for abdominal fat pad weight, which resulted in a 4.5-fold increase in the fat line compared to the lean line. Our analysis revealed that the proportions of *Fusobacteria* and *Proteobacteria* differed significantly between the two lines (8 vs. 18% and 33 vs. 24%, respectively) at the phylum level. Eight bacterial genera and 11 species were also substantially influenced by the host genotype. Differences between the two lines in the frequency of host alleles at loci that influence accumulation of abdominal fat were associated with differences in the abundance and composition of the gut microbiota. Moreover, microbial genome functional analysis showed that the gut microbiota was involved in pathways that are associated with fat metabolism such as lipid and glycan biosynthesis, as well as amino acid and energy metabolism. Interestingly, citrate cycle and peroxisome proliferator activated receptor (PPAR) signaling pathways that play important roles in lipid storage and metabolism were more prevalent in the fat line than in the lean line.

**Conclusions:**

Our study demonstrates that long-term divergent selection not only alters the composition of the gut microbiota, but also influences its functional performance by enriching its relative abundance in microbial taxa. These results support the hypothesis that the host and gut microbiota interact at the genetic level and that these interactions result in their co-evolution.

**Electronic supplementary material:**

The online version of this article (doi:10.1186/s12711-016-0270-5) contains supplementary material, which is available to authorized users.

## Background

The development of sequencing technologies for application in metagenomics has increased our capacity to investigate the composition and dynamics of the microbial communities that harbor diverse habitats [1]. The gastrointestinal tract is populated by a complicated and vast microbial network that influences the health and development of the host organism in numerous aspects [2, 3]. The gut microbial composition can be viewed as a polygenic trait, that not only produces essential products and forms a barrier against pathogens, but also has multiple functions in physiology, metabolism, immunity, development, and behavior of the host [4–6]. The gut microbiota causes the suppression of the circulating lipoprotein lipase inhibitor that results in increased lipoprotein lipase activity, which in turn results in a significant increase in body fat deposition in the host [7]. Suppression of the expression of these genes by direct action of the gut microbiota on the villi epithelia also causes increased lipoprotein lipase activity, which leads to increased triglyceride uptake and peripheral fat storage [8]. These findings are in agreement with previous studies in other chicken populations selected for high or low body fat [9, 10] and show that the gut microbiota affects energy uptake from the diet and energy storage in the host [7]. In our previous studies, in order to quantify the influence of genetic variation of the host on the structure of the gut microbiota, the abundance of gut microbiota was considered as a quantitative trait of the host, and we calculated the heritability of abundance of specific microorganisms in the gut microbiota. A few bacterial families of the microbiota had a moderate heritability, which indicated that the host genetics has an effect on the composition of the gut microbiota. Concurrently, we calculated the genetic correlations between specific microorganisms in the gut microbiota to examine if the genetics of the host is involved in the interactions between microorganisms in the gut microbiota. Significant genetic correlations between microorganisms in the gut microbiota were observed. Further analysis showed that such genetic correlations can be altered by genetic variation of the host. These results imply the importance of the host genetic background on the interactions between the microorganisms in the gut microbiota [11, 12]. However, the interactional mechanism between gut microbiota and genetic variation of the host genome has remained obscure. Until now, most studies focused on microbial taxa instead of microbial functional performance to understand the interactions between host genetics and gut microbiota.

Many factors influence the mechanism of the interactions between the host and the gut microbiota [13, 14]. Thus, choosing a model organism that is maintained in a controlled environment should enhance our understanding of the relationships between gut microbiota and host genetic factors. The chicken, which bridges the evolutionary gap between mammals and reptiles, serves as an important experimental model organism for the extant avian species due to the characteristics of its less complex gut microbiota and minimal maternal effect. Here, we analyzed and compared the function and classification of gut microbiota from two divergently selected lines of chickens, i.e. a fat line and a lean line. These lines originated from a single commercial grandsire line and underwent long-term (15 generations) divergent selection for abdominal fat percentage (AFP) and plasma very-low-density lipoprotein (VLDL) concentration. At 7 weeks of age, the mean adipocyte diameter in the fat line was 1.3 times wider than in the lean line, and the number of fat cells was 2.4 times larger in the fat line than in the lean line [15]. The long-term divergent selection also resulted in a 4.5-fold increase in abdominal fat pad weight in the fat line [16]. A total of 230 genes were found to be differentially expressed in the lean and fat lines; these genes are mainly related to signal transduction, tumorigenesis, immunity, and lipid and energy metabolism [17]. The two lines carry two main haplotypes with completely opposite single nucleotide polymorphism (SNP) alleles and a recombinant haplotype with nearly equal frequency in the 0.73-Mb *PC1*/*PCSK1* region of the Z chromosome. Genome-wide association analysis revealed that nearly all regions with evidence of selection signatures had SNP effects on abdominal fat weight and percentage [18].

## Methods

### Animals and samples collection

Two chicken lines (fat and lean lines) that were divergently selected for abdominal fat content (AFP) and plasma very-low-density lipoprotein (VLDL) were used in this study. Throughout all generations, they were maintained at the same location and reared on the same diets. Fecal samples were collected at 35 weeks of age from 29 fat line males, 26 lean line males, 27 fat line females, and 27 lean line females, for a total of 109 individuals from the 15th generation. The fecal samples were stored at −80 °C after collection. Animals were cared for in accordance with the Institute for Laboratory Animal Research (ILAR) guide for Care and Use of Laboratory Animals at Shanghai Jiao Tong University, China.

### Gut microbial 16S rDNA sequencing

Fecal microbial genomic DNA extraction and 16S rDNA amplification and sequencing were performed as previously reported in [11]. A QIAmp DNA Stool Mini Kit (Qiagen, cat#51504) was used for microbial genomic DNA extraction. Extracted DNA was measured using a nanodrop spectrophotometer (Thermo Fisher Scientific) to assess DNA quantity and quality. The V4 hypervariable region of the 16S rDNA gene was PCR-amplified from microbiota genomic DNA using sample-specific sequence barcode fusion primers (forward 5′AYTGGGYDTAAAGNG 3′, reverse 5′ TACNVGGGTATCTAATCC 3′). PCR reactions and PCR product purification were performed as previously reported in [11]. Purified PCR products from the 109 samples were combined at equal concentrations and used to construct a metagenomic library using Illumina TruSeq sample preparation kit (Illumina, USA) according to the manufacturer’s protocol. Sequencing was carried out by the Shanghai Personal Biotechnology Limited Company (Shanghai, P. R. China) using an Illumina MiSeq (Illumina, USA) sequencing platform. Sequence reads were quality-checked and removed based on the following criteria: reads that (1) contained ambiguous bases, (2) had an average phred score lower than 25, (3) contained a homopolymer run that exceeded 6, (4) contained mismatches in the primers, and (5) had sequence lengths that were outside the limits of 200 and 1000 bp. The filtered sequences with an overlap longer than 10 bp between Read 1 and Read 2 and without mismatches were assembled according to their overlapping sequences. Reads that could not be assembled were discarded. The barcode and sequencing primers were trimmed from each sequence.

### Analysis of classification and abundance

Based on the V4 region of the 16S rDNA sequence that passed the quality criteria, 2,301,532 amplicons were used for this study, with an average of 21,115 amplicons for each sample (ranging from 12,137 to 30,067) [see Additional file 1: Table S1]. The average sequenced amplicon length was 225 bp. Following filtering, each sample’s trimmed and filtered sequences were submitted to Metagenome Rapid Annotation using Subsystem Technology (MG-RAST) [19] and compared to the Ribosomal database project databases (RDP) [20] using the best hit classification option to classify the abundance count of each taxon. The metagenome sequences used in this paper are publicly available from MG-RAST under the project name “fatandleanchicken”. Data were generated at the species level, using cutoffs for the parameter classification at 8 for maximum e-value, 98% for minimum percentage identity, and 120 bp for minimum alignment length. A total of 37,590 taxa on the phylum, class, order, family, genus and species levels were annotated [see Additional file 2: Table S2]. Taxa that were present in at least 28 samples were considered as commonly existing classifications; 51 genera and 109 species met that criterion and their abundance counts were used for further analysis.

The taxon abundance counts were log2 transformed and normalized by subtracting the mean of all transformed values and dividing by the standard deviation of all log-transformed values for the given sample. After this procedure, the abundance profiles for all samples exhibited a mean of 0 and a standard deviation of 1. In order to detect if host genetic factors influence gut microbiota, *T* test was performed between fat and lean lines for specific microbes using Microsoft Excel, with adjustment of p values by Benjamini Hochberg FDR (FDR < 0.05) [21]. Alpha-diversity analysis was performed in mothur 1.31.2 [22] with the alpha-diversity.py script to calculate the index of chao1 and Shannon.

### Sequencing of the whole microbial genome

Microbial genomic DNA of three females from each fat and lean line was used to construct whole microbial genome sequencing libraries with insert sizes of 300 and 400 bp. Each library was sequenced by high-throughput sequencing at 2 × 100 bp using the Illumina HiSeq 2000 (Illumina, USA). Eighty percent of the whole microbial genome sequence data with paired-end Illumina sequences were accounted for across all samples. A data cleaning process was applied to all samples. Low-quality reads, and low-compositional-complexity reads were removed. An average of 37.9 million reads per sample were used in the analysis. The DNA sequences are publicly available in MG-RAST under the project name “Hiseqchicken-six”.

### Annotation of microbial function

Quality-filtered reads were submitted to MG-RAST and compared to the Kyoto Encyclopedia of Genes and Genomes (KEGG) database [23] using the ‘all annotation’ option for functional annotation with a maximum e-value cutoff of 1e-5, a minimum percent identity cutoff of 90%, and a minimum alignment length cutoff of 20 amino acids. Functional pathways, which had a relative abundance that was greater than 0.1% and for at least two samples, were chosen for further analysis. The relative abundance of each functional pathway was normalized within each sample. Clustering analysis was performed by Cluster 3.0 and Java Treeview [24] and differential analysis was evaluated by STAMP v2.0 [25] between fat and lean lines for abundance of each functional pathway by applying the two-side Welch’s t-test [26] and Benjamini-Hochberg FDR correction (FDR < 0.05).

## Results

### Diversity of gut microbial composition between the fat and lean lines

**Fig. 1:**
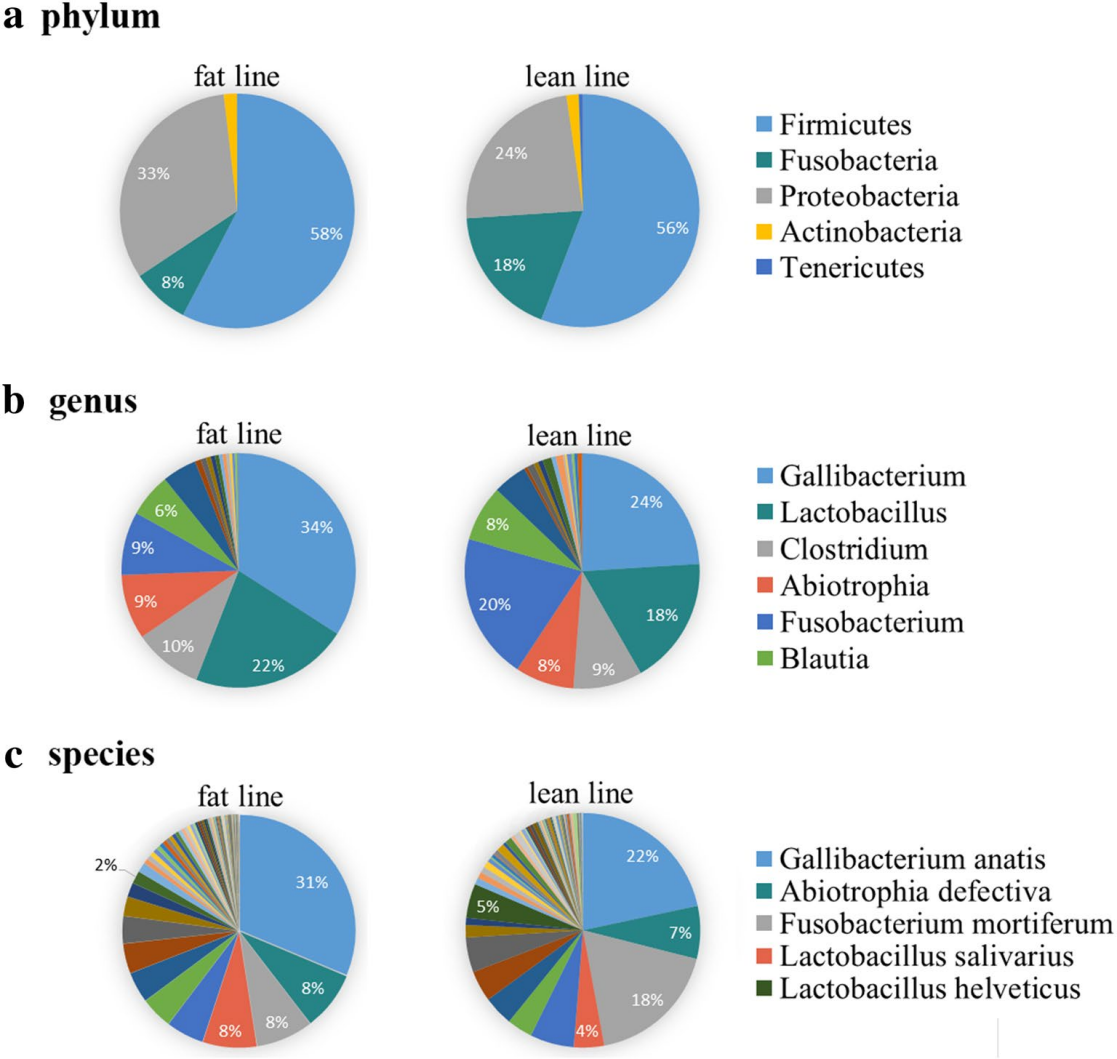
Aggregate microbiota composition at different levels in the fat and lean lines. **a** Phylum level, **b** genus level, **c** species level. Only major taxonomic groups are shown

16S rDNA amplicon sequencing was used to analyze the microbial diversity and abundance in the gut microbiota of the fat and lean chicken lines. Alpha diversity results suggested that the richness and diversity of gut microbiota were influenced by the long-term divergent selection [see Additional file 3: Figure S1]. Four major phyla dominated the chicken gut bacterial community; *Firmicutes* was the most predominant phylum, followed by *Proteobacteria, Fusobacteria,* and *Actinobacteria* (Fig. 1a). Consistent with previous studies related to avian microbial diversity, *Firmicutes* and *Proteobacteria* were the main ubiquitous members in the gut microbiota, but more *Fusobacteria* were classified compared with several other avian gut microbial studies [27–29]. The gut microbial composition differed between the fat and lean lines. The percentage of *Fusobacteria* was significantly lower in the fat line (8%) than in the lean line (18%). Conversely, the percentage of *Proteobacteria* was 33% in the fat line and approximately 24% in the lean line (Fig. 1a). At the genus level, *Gallibacterium,* which belongs to the phylum of *Proteobacteria* and comprises the *Gallibacterium anatis* species, was the most abundant genus in chicken gut microbiota (Fig. 1c). The genus *Fusobacterium,* belonging to the phylum of *Fusobacteria*, was in lower proportion in the fat line (9%) than in the lean line (20%) (Fig. 1b). The analysis showed that one (*Proteobacteria*) of the eight phyla [see Additional file 4: Table S3], eight of the 52 genera (Table 1) and 11 species [see Additional file 5: Table S4] were significantly influenced by the genetics of the host.

**Table 1:** Comparison of bacterial genus abundance in the gut microbiota between the fat and lean lines

Phylum	Genus	Relative fold change^a^ Fat versus lean line	*p* value (*p < 0.05, **p < 0.01)
*Actinobacteria*	*Rothia*	1.28	0.004**
	*Micrococcus*	1.20	0.009**
*Bacteroidetes*	*Bacteroides*	−1.08	0.019*
*Proteobacteria*	*Gallibacterium*	1.25	0.011*
*Tenericutes*	*Acholeplasma*	−1.19	0.03*
*Firmicutes*	*Aerococcus*	1.14	0.037*
	*Pectinatus*	−1.27	0.003**
	*Selenomonas*	1.17	0.04*

^a^+ fat/lean; − lean/fat

### The effect of host genetics on gene functional enrichment of gut microbiota

**Fig. 2:**
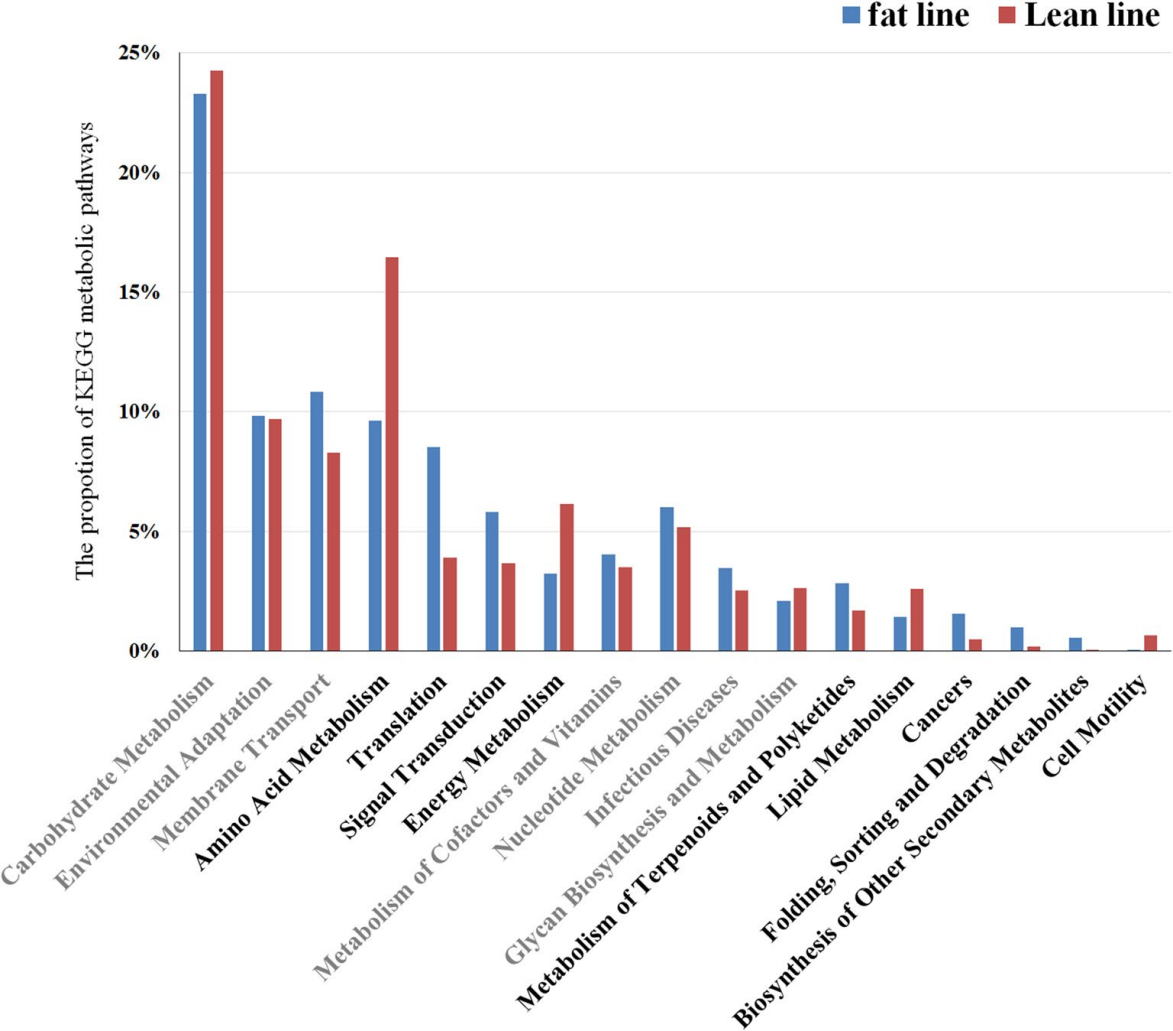
Distribution of KEGG metabolic pathways in the fat and lean lines. Profile *bar plots* show the relative proportion of each metabolic pathway. The pathways labeled in *black* were differentially expressed (fold change >1.5)

In order to investigate the influence of host genetic variation on the functional performance of the microbiota, we sequenced the whole gut microbial genome using three biological replicates from each line. Based on the associated KEGG orthologous group markers, we compared predicted microbial functions between the fat and lean lines and detected that amino acid metabolism, energy metabolism, lipid metabolism, and cell motility were nearly twofold more enriched in the lean line than in the fat line (Fig. 2). Pathways that were more enriched in the fat line included translation, signal transduction mechanisms, metabolism of terpenoids and polyketides, protein folding and degradation, biosynthesis of secondary metabolites, and cancers (Fig. 2). These results are consistent with the previous findings of a study on obese rats [30].

**Fig. 3:**
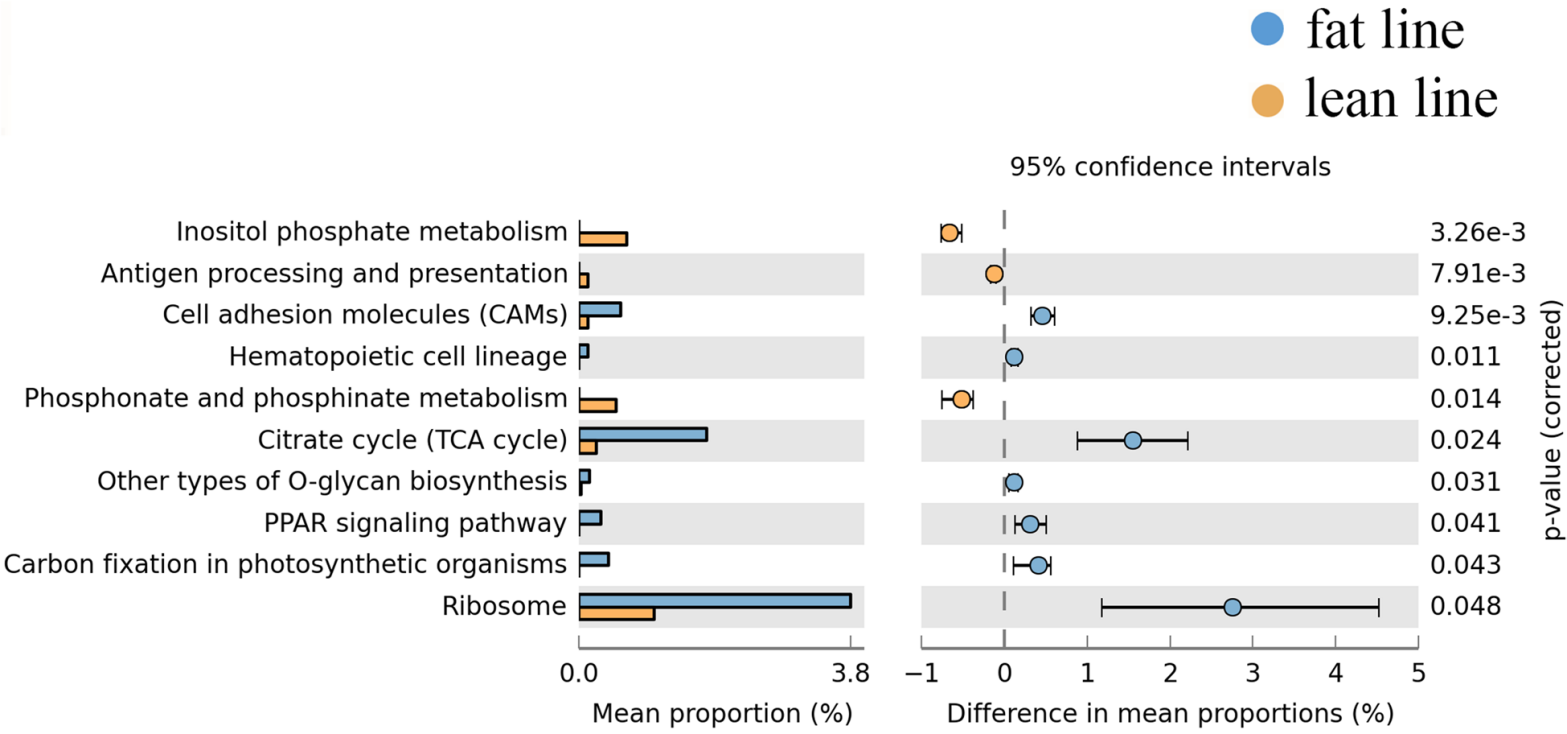
Significant differences in microbial metabolism pathways between the fat and lean lines

**Fig. 4:**
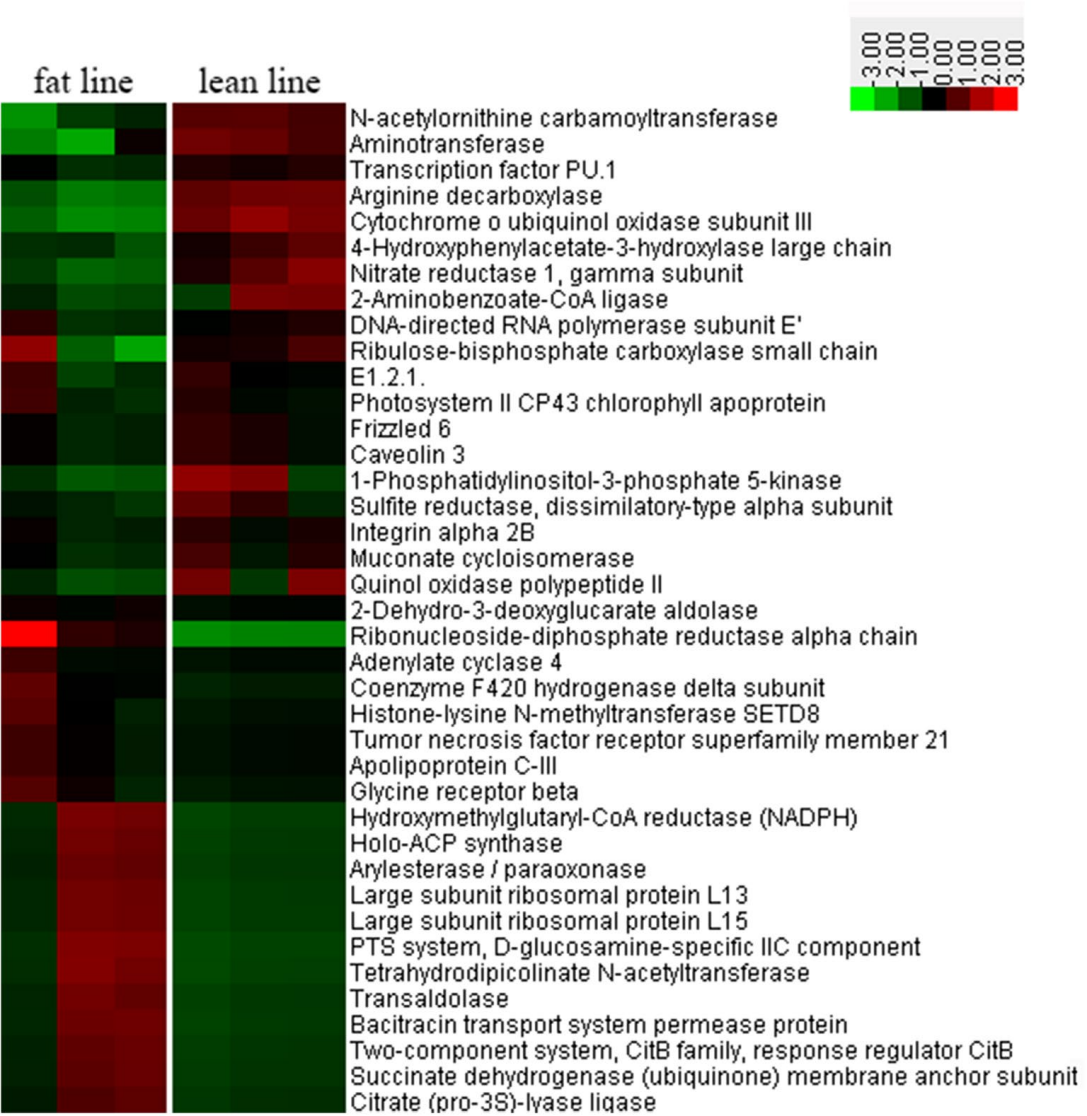
Heatmap of microbial function pathways in the fat and lean lines. *Colors* reflect relative abundance from low (*green*) to high (*red*); detailed categories for each gene are in Table S5 [see Additional file 6: Table S5]

Based on the analysis of whole microbial genome sequencing data, we observed that enriched markers were frequently involved in the functional pathways of inositol phosphate metabolism, antigen processing and presentation, and phosphonate and phosphinate metabolism in the lean line. In contrast, the gut microbiota of the fat line showed enrichment in citrate cycle, other types of o-glycan biosynthesis, peroxisome proliferator activated receptor (PPAR) signaling pathway, carbon fixation in photosynthetic organisms, ribosomes, and cell adhesion molecules (Fig. 3). A hierarchy cluster heatmap was generated to visualize the distribution of microbial functions in the fat and lean lines (Fig. 4). The microbial functions were also matched to the microbial metabolic pathway results from the study on obese mice [31]. The heatmap results suggested that aminotransferase, arginine decarboxylase, cytochrome o ubiquinol oxidase subunit III, and 1-phosphatidylinositol-3-phosphate 5-kinase, which are involved in amino acid metabolism, energy metabolism, and carbohydrate metabolism respectively, were more abundant in the lean line than in the fat line (Fig. 4) and [see Additional file 6: Table S5]. Compared to the lean line, the gut microbiota in the fat line had a higher functional performance related to bacitracin transport system permease protein, citrate (pro-3s)-lyase ligase, and ribonucleoside-diphosphate reductase alpha chain, which are respectively involved in immune system diseases, signal transduction and nucleotide metabolism (Fig. 4) and [see Additional file 6: Table S5].

## Discussion

Previous studies reported that the genetics of the host can influence the abundance and composition of gut microbiota. In this study, significant differences in 11 microbial species were observed between the fat and lean lines [see Additional file 5: Table S4]. Among these species, *Pectinatus frisingensis, Lactobacillus salivarius,* and *Micrococcus sp. SMCC ZAT352* were found to be associated with adipogenesis. For example, *P. frisingensis* synthesizes lipopolysaccharide with polymeric O-specific chains that are related to host obesity [32], *L. salivarius* can modify the fecal microbiota, which in turn affects metabolic pathways in obese chickens and humans [33, 34], and *Micrococcus* is involved in lipolytic activity, which shows a positive correlation with fatty acid biosynthesis [35]. Although 16S rDNA amplicon sequencing was the primary method used to analyze microbial diversity, we also used the computation tool PICRUSTs [36] to predict microbial community functions [see Additional file 7]. Functional prediction results revealed that signal transduction mechanisms and fatty acid biosynthesis were more abundant in the fat line than in the lean line and this was consistent with the results of the microbial composition of the gut microbiota [see Additional file 5: Table S4 and Additional file 8: Figure S2].

Several studies have shown that multiple transcription factors and signaling pathways are involved in the regulation of adipogenesis [37–39]. PPAR plays an important role in adipogenesis, adipocyte gene expression, and fat cell differentiation, which promote lipid storage and metabolism [40, 41]. Moreover, the PPAR signaling axis is also a potential target for the modulation of adipogenesis [42]. Interestingly, our whole microbial genome sequencing results suggested that, compared to the lean line, the PPAR signal pathway of gut microbiota in the fat line had a significantly higher functional performance (Fig. 4). This suggests the possibility that the PPAR signal pathway may also be involved in lipid storage. The citrate cycle is another key metabolic pathway that unifies carbohydrate, lipid, and protein metabolism. A significant correlation between citrate synthase level and obesity, together with a decreased activity of this enzyme in the mitochondria of human omental adipose tissue, were reported in obese humans [43]. Previous studies showed that citrate synthase activity was suppressed in obese mice, resulting in excessive carbon flow into the citrate cycle prompting energy storage [44]. Gut microbiota appears to play a key role in the development and progression of obesity, together with changes in citrate synthase activity [45, 46]. In this study, analysis of the results of whole microbial genome sequencing suggested that enrichment in the microbial function that relates to citrate cycle was significantly different between the fat and lean lines (Fig. 4). We have reasons to believe that microbes undertake many metabolic tasks, and that functional interactions between host genetic factors and gut microbiota are inevitable.

## Conclusions

We found that long-term divergent selection for abdominal fat has considerable influence on the abundance and composition of gut microbiota by altering the frequencies of obesity-related alleles. Furthermore, whole microbial genome sequencing results revealed that functional activities of the microbiota, such as those related to the citrate cycle and PPAR signaling pathway, differed significantly between the fat and lean lines and were affected by the gut microbiota and by differences in frequencies of host alleles. Our results provide further evidence for the hypothesis that host genetic factors interact and co-evolve with gut microbiota.

## Additional files

**Additional file 1: Table S1**. Summary of sequencing data for all samples.

**Additional file 2: Table S2**. Numbers of taxa at each level.

**Additional file 3: Figure S1**. Alpha diversity of bacteria in the gut microbiota of fat and lean lines. *Boxes* indicate the IQR (75th to 25th of the data). The median value is shown as a line within the *box* and the mean value as a *star*. Whiskers extend to the most extreme value within 1.5 * IQR. Outliers are shown as *crosses*. Higher chao1 suggests greater richness of microbes. Higher Shannon suggests greater diversity of microbes.

**Additional file 4: Table S3**. Comparison of microorganisms in the gut microbiota at the phylum level between the fat and lean lines. This table lists the relative fold change and *p* value of the differences in microorganisms at the phylum level between the fat and lean lines.

**Additional file 5: Table S4**. Comparison of microorganisms in the gut microbiota at the species level between the fat and lean lines. This table lists the relative fold change and *p* value of the differences in microorganisms in the gut microbiota at the species level between the fat and lean lines.

**Additional file 6: Table S5**. Corresponding metabolic pathways of the significant functions in the heatmap (related to Fig. 4). This table lists the corresponding metabolic pathways that are in Fig. 4.

**Additional file 7**. Prediction of microbial functions. This file describes the method for functional prediction based on 16S rDNA sequencing data.

**Additional file 8: Figure S2**. Predicted functional pathways in fat and lean lines based on 16S rDNA sequencing data.
