# Supplementary figures and images for: Divergent selection-induced obesity alters the composition and functional pathways of chicken gut microbiota
Source: Genet Sel Evol. 2016 Nov 28;48:93. doi: 10.1186/s12711-016-0270-5 (PMC5127100; doi:10.1186/s12711-016-0270-5)

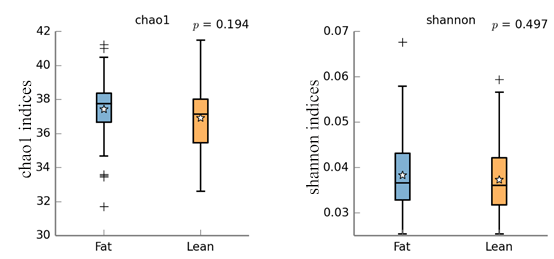

Supplement: Supplementary file 3 — Additional file 3: Figure S1. Alpha diversity of bacteria in the gut microbiota of fat and lean lines. Boxes indicate the IQR (75th to 25th of the data). The median value is shown as a line within the box and the mean value as a star. Whiskers extend to the most extreme value within 1.5 * IQR. Outliers are shown as crosses. Higher chao1 suggests greater richness of microbes. Higher Shannon suggests greater diversity of microbes. [file 12711_2016_270_MOESM3_ESM.tif]

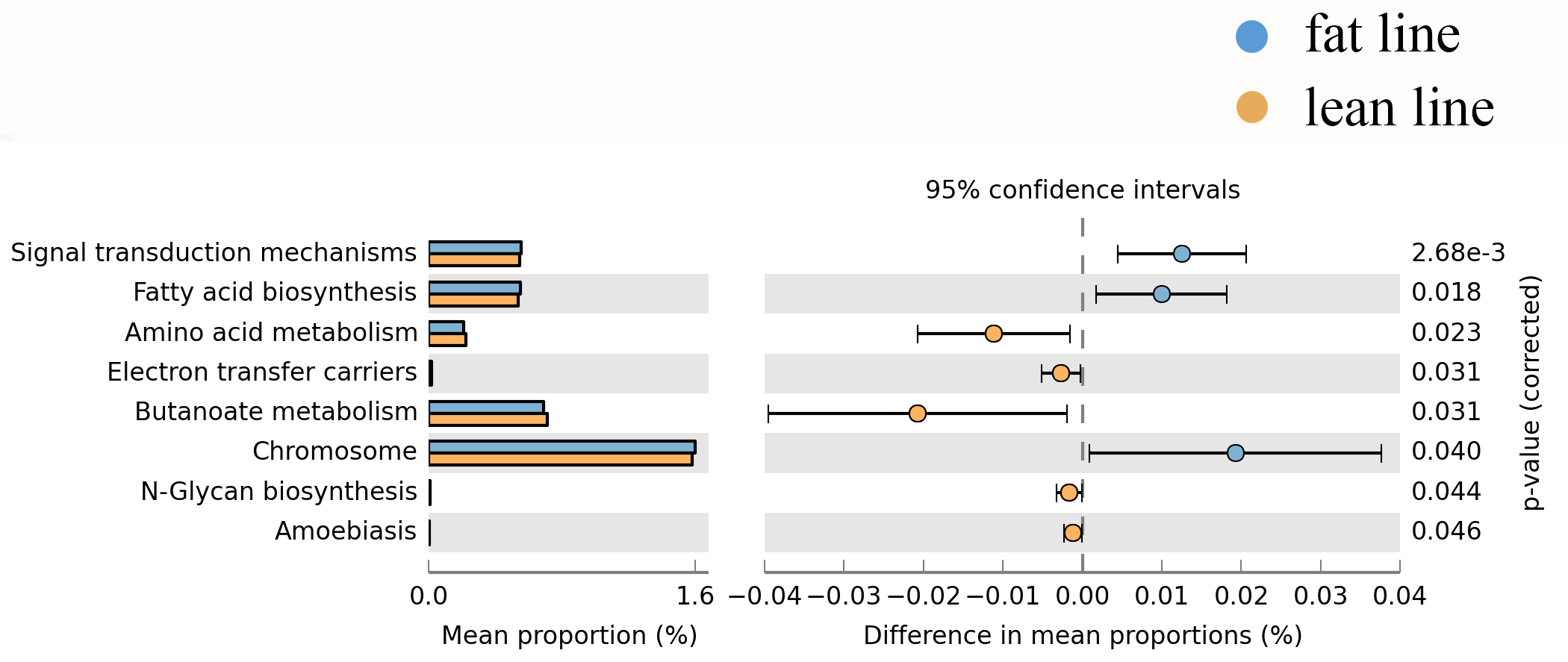

Supplement: Supplementary file 8 — Additional file 8: Figure S2. Predicted functional pathways in fat and lean lines based on 16S rDNA sequencing data. [file 12711_2016_270_MOESM8_ESM.tif]
