# Supplementary material for: Divergent selection-induced obesity alters the composition and functional pathways of chicken gut microbiota
Source: Genet Sel Evol. 2016 Nov 28;48:93. doi: 10.1186/s12711-016-0270-5 (PMC5127100; doi:10.1186/s12711-016-0270-5)
Supplement: Supplementary file 7 — Additional file 7. Prediction of microbial functions. This file describes the method for functional prediction based on 16S rDNA sequencing data. [file 12711_2016_270_MOESM7_ESM.docx]

**Additional file 7** Prediction of microbial functions

**Prediction of microbial functions**

The study of microbial communities is of little biological value unless the functional potential of the community, or individual members, are considered. Microbial function was predicted using PICRUSt. The taxa abundance were normalized automatically using 16S rDNA gene copy numbers from known bacterial genomes in Integrated Microbial Genomes (IMG). The predicted genes and their function were aligned to Kyoto Encyclopedia of Genes and Genomes (KEGG) database and the differences among groups were compared through software STAMP v2.0. Two-side Welch’s t-test and Benjamini-Hochberg FDR correction (FDR < 0.05) were used in two lines analysis.
